# Gene-based outcome prediction in multiple cohorts of pediatric T-cell acute lymphoblastic leukemia: a Children's Oncology Group study

**DOI:** 10.1186/1476-4598-9-105

**Published:** 2010-05-12

**Authors:** Amanda L Cleaver, Alex H Beesley, Martin J Firth, Nina C Sturges, Rebecca A O'Leary, Stephen P Hunger, David L Baker, Ursula R Kees

**Affiliations:** 1 Division of Children's Leukaemia and Cancer Research, Telethon Institute for Child Health Research, Perth, Australia; 2 Division of Biostatistics and Genetic Epidemiology, Telethon Institute for Child Health Research, Perth, Australia; 3 Centre for Child Health Research, University of Western Australia, Perth, Australia; 4 Department of Oncology & Haematology, Princess Margaret Hospital for Children, Perth, Australia; 5 Department of Pediatrics, University of Colorado, Denver, CO, USA; 6 The Children's Hospital, Aurora, CO, USA

## Abstract

**Background:**

Continuous complete clinical remission in T-cell acute lymphoblastic leukemia (T-ALL) is now approaching 80% due to the implementation of aggressive chemotherapy protocols but patients that relapse continue to have a poor prognosis. Such patients could benefit from augmented therapy if their clinical outcome could be more accurately predicted at the time of diagnosis. Gene expression profiling offers the potential to identify additional prognostic markers but has had limited success in generating robust signatures that predict outcome across multiple patient cohorts. This study aimed to identify robust gene classifiers that could be used for the accurate prediction of relapse in independent cohorts and across different experimental platforms.

**Results:**

Using HG-U133Plus2 microarrays we modeled a five-gene classifier (5-GC) that accurately predicted clinical outcome in a cohort of 50 T-ALL patients. The 5-GC was further tested against three independent cohorts of T-ALL patients, using either qRT-PCR or microarray gene expression, and could predict patients with significantly adverse clinical outcome in each. The 5-GC featured the interleukin-7 receptor (*IL-7R*), low-expression of which was independently predictive of relapse in T-ALL patients. In T-ALL cell lines, low *IL-7R *expression was correlated with diminished growth response to IL-7 and enhanced glucocorticoid resistance. Analysis of biological pathways identified the NF-κB and Wnt pathways, and the cell adhesion receptor family (particularly integrins) as being predictive of relapse. Outcome modeling using genes from these pathways identified patients with significantly worse relapse-free survival in each T-ALL cohort.

**Conclusions:**

We have used two different approaches to identify, for the first time, robust gene signatures that can successfully discriminate relapse and CCR patients at the time of diagnosis across multiple patient cohorts and platforms. Such genes and pathways represent markers for improved patient risk stratification and potential targets for novel T-ALL therapies.

## Background

T-cell acute lymphoblastic leukemia (T-ALL) affects approximately 15% of newly diagnosed pediatric ALL patients. Continuous complete clinical remission (CCR) in T-ALL patients is now approaching 80% due to the implementation of aggressive chemotherapy protocols [1-6]. However, patients that relapse (R) have poor prognosis and aggressive therapy can lead to long-term side effects in those that achieve CCR [7]. In the clinical setting, age and white blood cell count (WBC) at diagnosis are used to stratify B-lineage ALL patients as either standard or high risk, significantly impacting on the type and intensity of post-induction therapy used. However these NCI-defined criteria have been shown to have little prognostic value in T-ALL disease [1-3]. Improved markers are needed for outcome prediction to improve T-ALL patient stratification.

Common karyotypic abnormalities have been identified in some forms of leukemia and have proven useful for outcome prediction [8-12]. In precursor B-lineage ALL (pre-B ALL), the presence of hyperdiploidy or translocations such as *E2A-PBX1*, *BCR-ABL*, or *ETV6-RUNX1 *contribute to the severity of disease and response to chemotherapy [8,9]. In T-ALL, increased expression of *TLX1/HOX11 *has been associated with favorable outcome [10,11,13,14], whilst aberrant expression of *TAL1*, *LYL1 *and *TLX3 *and deletions at 6q15-16.1 have been linked to poor prognosis [11,15,16]. Recent work by Coustan-Smith and colleagues [17] has led to the identification of a new very high risk subset of T-ALL (early T-cell precursor leukemia) that has a distinct expression profile and immunophenotype. However, due to the lack of consensus between studies and the small proportion of T-ALL patients that carry these genetic or molecular aberrations, the identification of a universal molecular signature has become a priority. Several studies have attempted to identify gene signatures that predict induction failure and/or relapse in T-ALL [8,18,19], but have had limited success verifying their findings in other patient cohorts. The current study aimed to identify robust gene signatures that could be used for the accurate prediction of relapse at the time of diagnosis, in independent patient cohorts, and across different experimental platforms.

## Materials and methods

### Patients

The study cohort comprised 84 T-ALL patients treated on Children's Oncology Group (CCG/COG) protocols (1882 - 1961) for high risk ALL [4]. Bone marrow specimens were obtained at diagnosis from patients at the Princess Margaret Hospital, Perth, Australia (n = 8) or COG (n = 76). Ethical approval was obtained from the Institutional Review Boards, and informed consent for the use of tissues was obtained for all individuals. These specimens were assigned to either Training (n = 50) or Validation (n = 34) Cohorts, based on amount of material available for microarray and/or quantitative RT-PCR (qRT-PCR) experiments. Clinical features of these cohorts are shown in Table 1. All patients achieved remission following induction therapy; those patients achieving complete continuous remission (CCR) had median follow-up times of 7.3 years (Training Cohort) and 8.8 years from diagnosis (Validation Cohort). 44% of the patients in the Training Cohort and 27% in the Validation Cohort subsequently relapsed (R).

**Table 1:** Clinical features of T-ALL patients in the Training and Validation Cohorts

	Training cohort (n = 50)		Validation cohort (n = 34)	
	**CCR (n = 28)**	**Relapse (n = 22)**	**CCR (n = 25)**	**Relapse (n = 9)**

**Sex**				
Male/Female	21/7	21/1	14/11	9/0
**Age at diag (years)**				
Median (Range)	13.1 (2.1-16.9)	12.1 (1.8-17.8)	7.1 (2.2-18.3)*	8.8 (1.8-17.5)
**WBC (×10^9^/L)**				
Median (Range)	171.9 (1.1-791)	219.2 (4.9-700)	113.1 (8.2-524.4)	161.8 (13.4-882)
**BM blast at diag (%)**				
Median (Range)	94 (70-100)	91 (74-99)	90 (35-99)	95 (70-99)
**Cytogenetics**				
Normal (46 C)	2	3	13	4
Pseudodiploid (46 C)	12	6	5	2
Hyperdiploid (>47 C)	3	2	3	0
Hypodiploid (<46 C)	0	0	2	1
N/A	11	11	2	2
**NCI Risk**				
Standard	0	0	6	1
High	28	22	19	8
**Induction result**				
M1	25	19	24	8
M2	3	0	0	0
M3	0	0	0	0
N/A	0	3	1	1
**Follow-up time (years)**				
Median (Range)	7.3 (3.3-9.2)		8.8 (4.3-11.9)	
**Time to relapse (years)**				
Median (Range)		1.3 (0.2-3.8)		1.4 (0.5-3.3)

WBC, white blood cell count; BM, bone marrow; diag, diagnosis; C, chromosomes; N/A, not available; NCI, National Cancer Institute; M1, <5%blasts in BM; M2, 5-25% blasts in BM; M3, >25% blasts in BM. *P = 0.0082 by Mann-Whitney t-test compared to CCR in the training cohort.

### Gene expression profiling

RNA from the T-ALL Training Cohort (n = 50) was extracted from bone marrow specimens and hybridized to HG-U133Plus 2.0 GeneChips (54,675 probe sets; Affymetrix, Santa Clara, CA, USA) according to Affymetrix protocols. Gene expression data were extracted and normalized using robust multi-array analysis (RMA) [20] as previously described [21-23]. Expression data from the two Winter *et al *cohorts (microarray CEL files and patient details) were obtained from the authors [19] and normalized by RMA. Induction failure cases were removed prior to analysis, resulting in cohort sizes of 44 patients for Pediatric Oncology Group (POG/COG) Protocol 9404 (30 CCR, 14R, measured on HG-U133 Plus 2.0 arrays) and 41 patients for POG 8704 (24CCR, 17R, measured on HG-U133A arrays). The modeling approaches used to develop gene-classifiers (GCs) from microarray data is described in the results section. For outcome prediction using the obtained GCs, logistic regression was used to model probability of relapse for each specimen based on gene expression scores (microarray data or qRT-PCR data as relevant). This probability was used as a continuous variable in Cox proportional hazard regression analysis, and converted into a prediction of CCR/relapse labels using a probability cut-off point of 50%, generating model accuracies, sensitivity, specificity, positive predictive values (PPV, the proportion of patients among those predicted to relapse that actually relapsed) and negative predictive values (NPV, the proportion of patients among those predicted as non-relapse that actually achieved CCR). This stratification was also used for Kaplan-Meier survival analysis, with significance determined by log-rank test.

### Real-time quantitative RT-PCR (qRT-PCR)

qRT-PCR was performed using TaqMan Gene Expression Assays (Applied Biosystems, Foster City, CA, USA) as previously described [21]. Reactions were performed in duplicate and run on an ABI Prism 7000 sequence detector (Applied Biosystems). The *ACTB *gene was used for normalization and standard curves were utilized for the quantitation of target gene expression.

### Effect of IL7 on cell growth and drug sensitivity

The features of the T-ALL cell lines and culture conditions used in this study have previously been described [24,25]. RNA for qRT-PCR was extracted from cell lines in log-phase growth. Surface expression of IL-7Rα (CD127) was assessed using PE-conjugated anti-human monoclonal IL-7Rα antibody (Immunotech, Marseilles, France) and an LSR II flow cytometer (BD Biosciences). The 3-(4,5-dimethylthiazol-2-yl)-2,5-diphenyltetrazolium-bromide (MTT) assay was used to determine growth responses to human recombinant IL-7 (R&D Systems, Minneapolis, MN) according to our published methods [24].

## Results

### Modeling a multi-gene classifier for outcome prediction

**Figure 1:**
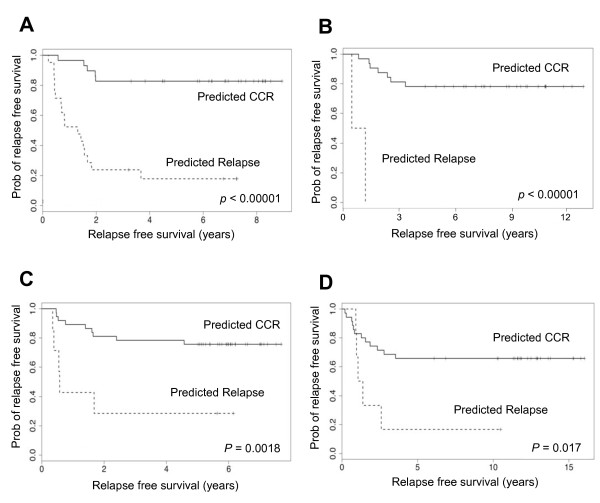
**Kaplan-Meier survival curves for patients predicted as CCR or relapse using the 5-GC model in (A) Training Cohort (n = 50); (B) Validation Cohort (n = 34); (C) COG 9404 (Winter *et al*, n = 44); (D) POG 8704 (Winter *et al*, n = 41)**.

Using the decision-tree based algorithm Random Forest (RF) as previously described [23,26], we ranked the 54,675 probe sets on the HG-U133 Plus 2.0 GeneChip for their ability to distinguish the 22 R and 28 CCR patients from the Training Cohort. Starting with the top-ranked 500 probes from this RF analysis we firstly filtered out probe sets derived from the HG-U133B predecessor GeneChip (which largely target expressed sequence tags and non-confirmed gene content), then applied a previously developed algorithm to identify genes with a high probability of detection by qRT-PCR [21]. This shortlist of 57 probe sets (Table S1, Additional file 1) was then used to model the optimal combination of 5 genes (5 gene classifier, 5-GC) for outcome prediction in the Training Cohort. We sought a small classifier of this size to facilitate its translation into clinical laboratories for routine testing at diagnosis. Principle Component Analysis (PCA) was used to rank gene combinations, based on the ability of the 1^st ^principal component to explain the variability between R and CCR patient specimens (r^2^). BLAST searches were performed to confirm that probe sequences were specific for the annotated gene specified, and TaqMan probes for corresponding sequences were identified. Logistic regression was used to assess the accuracy of R/CCR status prediction for each 5-GC. In this way we identified a 5-GC that could predict R/CCR outcome in patients from the Training Cohort (n = 50) with an overall accuracy of 82% (model performance, p < 0.0001; Fisher's Exact Test). These genes were *ABTB2*, *IL7R*, *LGALS8*, *PLAC8*, and *FAM13A1 *(Table 2). Kaplan-Meier analysis demonstrated that patients labeled as R and CCR using gene expression scores of the 5-GC had significantly different relapse-free survival times (Figure 1A, p < 0.0001). Cox proportional regression analysis of age, WBC, gender and 5-GC score demonstrated that the 5-GC score was the most significant factor related to outcome (p < 0.0001, Table 3). The fact that age, WBC and gender were not significantly associated with outcome in the present study is a reflection of the size of the cohort involved and emphasizes the need for additional prognostic markers. Expression of the 5-GC was also measured by qRT-PCR in 40 specimens from the Training Cohort for which sufficient material was available. Table 2 summarizes the expression levels and R/CCR fold-changes for the identified genes, measured by both microarray and qRT-PCR. The recorded fold-changes were comparable between microarray and qRT-PCR data and the data correlated significantly between the two techniques (p < 0.001) for all genes (Pearson's correlation, log_2 _values).

**Table 2:** Expression of genes from the 5-GC measured by both array (HG-U133Plus2) and qRT-PCR in specimens from the Training Cohort

Gene Symbol (Probe ID)	Gene	RF Rank	Mean Expression (Array)		Fold Change (R/CCR)		r
				
			R	CCR	Array	qRT-PCR	
ABTB2 (213497_at)	Ankyrin repeat and BTB (POZ) domain-containing 2	12	119.8	89.7	1.34	3.11	0.76
IL7R (205798_at)	Interleukin-7 receptor	43	292.0	542.7	0.54	0.47	0.92
LGALS8 (208936_x_at)	Lectin, galactose binding, (Galectin 8)	288	197.4	231.8	0.85	0.37	0.56
PLAC8 (219014_at)	Placenta-specific 8 (Onzin)	297	530.4	979.5	0.54	0.22	0.90
FAM13A1 (217047_s_at)	Family with sequence similarity 13, member A1	356	70.0	84.0	0.83	0.30	0.69

r, correlation between HG-U133Plus2 and qRT-PCR fold change data (n = 40); R, relapse; CCR, continuous complete remission.

**Table 3:** Univariate Cox proportional hazard regression analyses of the risk of relapse in the Training Cohort (n = 50) in relation to diagnostic features and the 5-GC score

Variable	No. of Patients	Hazard Ratio	**95% CI **^**a**^	p-value
**Age at diagnosis**				
<10 years	17	1 ^b)^		
≥10 years	33	1.3	(0.506, 3.32)	0.59
**WBC**				
<50/nl	12	1 ^b)^		
>50/nl	38	1.15	(0.425, 3.12)	0.78
**Gender**				
Female	8	1 ^b)^		
Male	42	5.53	(0.742, 41.2)	0.095
**5-GC score**	50	1.31	(1.19, 1.44)	<0.0001

a) 95% confidence interval; b) reference group.

### Validation of the 5-GC in an independent cohort

We subsequently determined the expression levels of the genes in the 5-GC by qRT-PCR in diagnostic bone marrow specimens from 34 pediatric T-ALL patients from a completely independent Validation Cohort. Most of these patients were treated on COG 1882 or 1901, while the patients in the Training Cohort were treated on COG 1961 [4]. In the Validation Cohort the 5-GC yielded an overall prediction accuracy of 79% (Table 4). Whilst model-performance was borderline by Fisher's Exact Test (*p *= 0.064, Table 4), Kaplan-Meier analysis demonstrated significant differences in relapse-free survival (Figure 1B, p < 0.0001). Although there was low sensitivity with only 2/9 relapsing patients successfully identified, PPV and specificity were 100%, meaning that no patients were incorrectly identified as relapsers. Cox proportional regression analysis confirmed the 5-GC score to be a significant factor related to outcome in this cohort (p < 0.05, Table S2, Additional file 1).

**Table 4:** Validation of gene-classifier models for outcome prediction across multiple T-ALL cohorts

Model	Cohort	Acc	PPV	NPV	Sens	Spec	*P*-value
5-GC (5 genes)	Training	82	81	83	77	86	9.4 × 10^-6^
	Validation	79	100	78	22	100	0.064
	COG 9404†	75	71	76	36	93	0.025
	POG 8704†	68	83	66	29	96	0.066
	**Combined**	**76**	**81**	**75**	**47**	**93**	**1.5 × 10^-9 ^(1.2 × 10^-4^)**

Pathway NFκB (7 genes)	Training	76	71	81	77	75	4.8 × 10^-4^
	Validation	-	-	-	-	-	-
	COG 9404†	77	83	76	36	97	0.009
	POG 8704†	56	44	59	24	79	1
	**Combined**	**70**	**67**	**72**	**49**	**84**	**7.2 × 10^-5 ^(0.073)**

Pathway Wnt/Ca^2+^/cGMP (12 genes)	Training	76	75	77	68	82	4.6 × 10^-4^
	Validation	-	-	-	-	-	-
	COG 9404†	75	67	77	43	90	0.019
	POG 8704†	68	63	72	59	75	0.05
	**Combined**	**73**	**69**	**76**	**58**	**83**	**8.7 × 10^-7 ^(0.0011)**

Pathway Cell Adhesion (14 genes)	Training	82	84	81	73	89	8.6 × 10^-6^
	Validation	-	-	-	-	-	-
	COG 9404†	75	62	81	57	83	0.012
	POG 8704†	85	87	85	76	92	1.1 × 10^-5^
	**Combined**	**81**	**79**	**82**	**70**	**88**	**8.2 × 10^-12 ^(4.5 × 10^-7^)**

Cohorts represent the Training Cohort (HG-U133Plus2, n = 50), Validation Cohort (qRT-PCR, n = 34), COG 9404 (HG-U133Plus2, n = 44), and POG 8704 (HG-U133A, n = 41); Acc, Accuracy; PPV, Positive Predicted Value; NPV, Negative Predicted Value; Sens, Sensitivity; Spec, Specificity (all model performances given as % and combined using means weighted for cohort size and CCR/relapse patient composition); *P*-value, Fisher's Exact Test of individual or combined model performances across the four cohorts (significance with the omission of the Training Cohort given in parenthesis); † Microarray cohorts downloaded from Winter *et al *(2007).

### In silico verification across multiple platforms and studies

A number of other studies have attempted to find outcome predictors in pediatric T-ALL using gene expression at the time of diagnosis. Yeoh, *et al*[8] identified seven genes (*UQCRFS1*, *SMA5*, *PRPSAP2*, *NCAPD3*, *TXBAS1*, *HMRPH2 *and *CD44*) as being differentially expressed between R and CCR in a cohort of 37 T-ALL patients using U95Av2 arrays. Our own group has previously identified three genes, *CFLAR*, *NOTCH2 *and *BTG3*, as prognostic markers in a smaller study of 12 T-ALL patients using HG-U133A arrays [18]. None of these previously identified prognostic markers featured in the top list of informative genes from the present study. In a different approach, Ferrando and colleagues [11] have demonstrated the predictive value of molecular signatures linked to oncogene expression (*TLX1, TAL1, LYL1, LMO1*, and *LMO2*) in T-ALL. However, the expression of these individual oncogenes did not significantly predict outcome in the present study. These observations demonstrate the difficulty of developing gene-based classifiers that can predict outcome across multiple platforms and patient cohorts.

In a recently published article, Winter *et al*[19] used HG-U133-Plus 2.0 GeneChips in pediatric T-ALL patients with the aim of identifying a gene signature in diagnosis specimens that could be linked to relapse or induction failure. The study comprised a cohort of 50 patients treated on therapy protocol COG 9404 and a cohort of 42 patients treated on POG 8704, but was unable to identify a gene signature to distinguish R from CCR cases. However, a gene signature linked to induction failure was identified. Comparison of this induction failure signature with our 500 top-ranked genes in the present study indicated just one gene in common, hexokinase II, which ranked #101 in the Winter *et al *gene list and #65 in our list and was upregulated in patients that went on to relapse. This gene is of interest because of its role in coordinating metabolic and apoptotic pathways at the mitochondrial membrane, and its reported association with glucocorticoid resistance in T-ALL [25]. To verify our own gene classifier in the two cohorts studied by Winter *et al*, we downloaded the array data from their study, removed the induction failure cases, and applied the 5-GC in logistic regression to predict R/CCR status in the remaining patients. The 5-GC predicted patient outcome with an overall accuracy of 75% in the COG 9404 cohort, and 68% in the POG 8704 cohort (Table 4). Importantly, patients predicted to relapse by the 5-GC had significantly worse survival rates in both cohorts (p < 0.05 for both, Figure 1C &1D). The weighted averages for the model performance across the four cohorts are summarized in Table 4 along with a combined p-value (generated by global assessment of performance accuracies in each independent cohort). Whilst the p-values for model performance were borderline in the three test-back cohorts individually, the consistent performance over all four cohorts was highly significant (p < 0.0001, Table 4). This combined analysis remained highly significant when the results from the original Training Cohort were excluded (p < 0.0005). Taken together these data indicate that the 5-GC has prognostic relevance across four independent T-ALL patient cohorts.

### Functional relevance of the IL-7R as a prognostic marker

**Figure 2:**
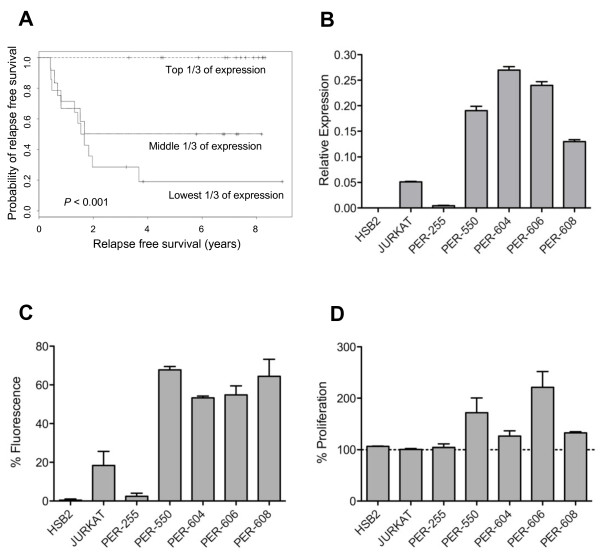
**Functional relevance of *IL-7R *as a prognostic marker**. (A) Kaplan-Meier survival curves based on levels of *IL-7R *(qRT-PCR mRNA expression tertiles) in the Training Cohort; (B) *IL-7R *mRNA expression in a panel of T-ALL cell lines measured by qRT-PCR; (C) Cell surface IL-7R (CD127) protein expression in T-ALL cell lines measured by flow cytometry; (D) Growth response of T-ALL cell lines over 4 days to exogenous IL-7 (10 ng/ml) as measured by MTT (% proliferation compared to medium control).

Of the genes identified as part of the 5-GC, the *IL-7R *is of particular relevance to T-ALL since IL-7 is known to be a key regulator of T-cell development [27]. Kaplan-Meier analysis in the Training Cohort demonstrated that low expression of the *IL-7R*, as a single variable, was significantly predictive of adverse outcome (p < 0.001, Figure 2A). To assess the functional significance of variations in *IL-7R *expression, we studied seven T-ALL cell lines [24], assessing *IL-7R *mRNA expression by qRT-PCR (Figure 2B) and cell surface expression by flow cytometry (Figure 2C). A significant correlation was demonstrated between mRNA and protein expression in the cell lines (p < 0.05), with some lines demonstrating little or no IL-7R expression (HSB2, JURKAT, PER-255). To examine the relationship between *IL-7R *expression and drug sensitivity we examined microarray gene signatures that we have previously correlated with glucocorticoid resistance patterns in an extended panel of 15 T-ALL cell lines [25], that includes those shown in Figure 2. Across the 15 lines there was a significant inverse correlation between mRNA expression of the *IL-7R *and IC50 scores to both dexamethasone (r = -0.673; p < 0.01) and methylprednisolone (r = -0.631, p < 0.02, Pearson's correlations), such that low *IL-7R *mRNA expression corresponded to glucocorticoid resistance. Thus the mean IC50 for the low *IL-7R *expressing lines in Figure 2C (HSB2, JURKAT, and PER-255) is three orders of magnitude higher (167 μg/ml DEX; 259 μg/ml MPRED) than for the four high expressing lines (0.03 μg/ml DEX; 0.06 μg/ml MPRED). Figure 2D demonstrates that only lines expressing *IL-7R *at relatively high levels respond to the addition of exogenous recombinant IL-7. Together these data are consistent with previous observations, made using primary T-ALL specimens, that IL-7 non-responsiveness correlates with reduced response to glucocorticoid therapy, and is thus an adverse prognostic indicator [28].

### Outcome prediction modeling using enriched biological pathways

**Figure 3:**
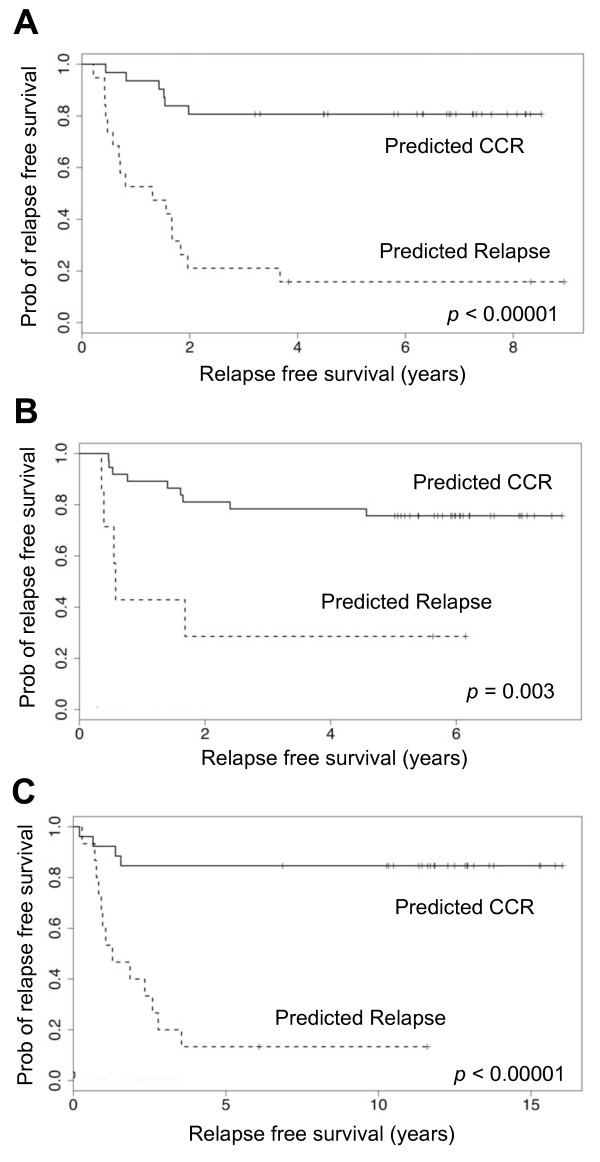
**Kaplan-Meier survival curves for patients predicted as CCR or relapse using the 14 gene 'Cell Adhesion Receptor' biological model in (A) Training Cohort (n = 50); (B) COG 9404 (Winter *et al*, n = 44); (C) POG 8704 (Winter *et al*, n = 41)**.

The 5-GC model was developed through a process of statistical, rather than biological, modeling to generate a robust diagnostic classifier. Although the individual genes that comprise the 5-GC have links to cancer biology and tumor development (see Discussion) we do not propose that they represent a coherent biological signature to explain clinical relapse. As a complementary approach therefore, we turned to Gene Set Enrichment Analysis (GSEA, http://www.broad.mit.edu/gsea) [29] to gain insight into the biological pathways differentially regulated in R vs. CCR specimens. Analysis was performed using the ranked gene list from the RF performed in the Training Cohort (focusing on HG-U133A probesets only), and three biological gene sets were identified at the relevant false discovery rate (FDR) < 25% and nominal p-value < 0.001. These were (i) NFκB pathway regulated genes, (ii) Genes of the Wnt/Ca^2+^/cGMP pathway (Global Cancer Map, Broad Institute), and (iii) Genes with cell adhesion receptor activity (Global Cancer Map, Broad Institute). The genes from these pathways that contribute most to the observed phenotype are referred to as the 'leading edge' and are listed in Table S3, Additional file 1. These leading edge genes were used in logistic regression to model outcome based on expression of each of the three pathways. Outcome prediction using the NFkB-pathway or Wnt/Ca^2+^/cGMP pathway models returned accuracies of >75% in both the Training Cohort, and the COG 9404 cohort, but did not perform so strongly in the POG 8704 cohort (Table 4). However, the cell adhesion receptor pathway model accurately predicted outcome (75-85%) in all three cohorts (Table 4). These performances were significant in each of the individual cohorts and highly significant in the global analysis (p < 0.0001, Table 4). Kaplan-Meier plots demonstrated that patients predicted to relapse using this model had significantly reduced relapse-free survival times in all three cohorts (Figure 3).

## Discussion

The fate of children who relapse with T-ALL remains dismal. This has fuelled considerable research into the discovery of 'risk factors' that are indicative of a patient's likelihood of relapse before post-induction therapy is prescribed. Our study statistically modeled a 5-GC that successfully predicted T-ALL patient outcome in four independent studies across different platforms. In a complementary approach we used biological signatures to successfully model patient outcome in these studies. To our knowledge this is the first time gene classifiers have been developed that accurately model ALL relapse in more than two independent cohorts. It is important to note that the models described here could not be used to down-grade a patient's risk classification since predictions of outcome under these models is in the context of the therapy each patient actually received. Thus CCR patients in these cohorts may potentially have relapsed if treated with lesser therapy. However, it would be possible to use such models to augment therapy. In the case of patients already stratified as high-risk (the majority of T-ALLs), this could include bone marrow transplant in first remission [6] or the use of experimental therapeutics. The best models generated in this study (the 5-GC and Cell Adhesion Pathway) were associated with good average specificity across the four cohorts (88-93%), but achieved lower average sensitivity (47-70%). In clinical terms this means that application of these models as a diagnostic test could have successfully identified up to two-thirds of the patients in these cohorts destined to relapse, whilst potentially over-treating only a small percentage of patients (7-12%) that would have achieved CCR under current protocols. Although higher sensitivity would be desirable, the correct identification of even a few patients destined to relapse could further improve cure rates.

In recent years much criticism has been directed towards microarray studies aiming to identify gene markers from small cohorts. Owing to the dimensionality of the data it is often possible to select genes at random that can discriminate between two phenotypes or patient subgroups with surprising accuracy. Furthermore, many statistical tools over-fit data such that the ability of classifiers to discriminate between phenotypes only extends to the cohort in which they were developed. However the probability of selecting gene classifiers at random that can discriminate between phenotypes in more than one cohort is vanishingly small. Validation of classifiers across multiple cohorts as described here (especially those identified using a permutative resampling algorithm such as the Random Forest) is empirical evidence of their robustness.

In this study low expression of the *IL-7R *was recorded in diagnostic T-ALL specimens from patients who later relapsed, linking low *IL-7R *expression to eventual therapy failure. The IL-7 cytokine is normally essential for T-cell development, survival and proliferation [27], and can inhibit both spontaneous apoptosis [28] and the apoptotic responses to chemotherapeutic agents in T-ALL [30], with the level of expression of the *IL-7R *correlating with these responses [28]. As such, IL-7 has been proposed as an important factor supporting leukemogenesis [31], but a proportion of T-ALL patients have blasts that do not respond to IL-7 [28]. This latter observation has been correlated with tumor maturation stage but it is also possible that it represents the acquisition of growth-factor independence. Growth-factor independence is a classical hallmark of a successful cancer cell [32] and indicates the development of potent pro-survival mechanisms. Importantly, T-ALL patients with an IL-7 non-responsive phenotype demonstrate poorer clinical responses to glucocorticoid therapy and thus have an adverse prognosis [28], consistent with our own findings in the present study.

Despite the obvious relevance of the IL-7R for T-ALL, the genes of the 5-GC were not selected on the basis of biological function. As such the 5-GC is considered as a tool for prognosis rather than for the interpretation of mechanisms of relapse, although the individual genes themselves do have links with cancer. The *ABTB2 *gene is involved with protein-protein interactions through its ankyrin repeats and BTB (POZ) domains. Although its specific function is unknown, one study has reported the up-regulation of *ABTB2 *in gastric tumor metastasis, highlighting the possible role of this gene in aggressive malignant phenotypes [33]. The *FAM13A1 *gene has an unknown function but is induced in various cell types exposed to hypoxic conditions [34]. It has been reported to be down-regulated in malignant thyroid tissue [35] but up-regulated in ovarian and breast cancer with links to poor prognosis [34]. *PLAC8 *is conserved in all vertebrates and is expressed at high levels in immune cell types [36]. The function of this gene is also unclear but has been linked to proliferation and apoptosis. *PLAC8 *is over-expressed in hepatocellular carcinoma tumours [37] and reduced in Paclitaxel-resistant prostate cancer [38].

Our alternative approach focused on identifying biological pathways that are involved in the progression to therapy failure in T-ALL. The NFκB and Wnt signaling pathways both had significant predictive power in this regard. The NF-κB pathway is highly active in T-ALL and is one of the major mediators of *NOTCH1*-induced transformation, establishing NFκB as a potentially promising target for T-ALL therapy [39]. The Wnt pathway is also important for T cell development and proliferation and is deregulated in several types of leukemia [40]. Although few studies have directly reported a role for Wnt signaling in the pathogenesis of T-ALL, antagonism of Wnt signaling has been shown to lead to chemotherapy resistance in a model of acute myeloid leukemia, *via *the downstream action of NFκB [41]. The pathway model that predicted relapse with the highest accuracy across all four cohorts in the present study was the Cell Adhesion Receptor geneset, with 12 out of the 14 genes representing integrins (Table S3, Additional file 1). Interestingly, *LGALS8*, the final member of the 5-GC, codes for a secreted mammalian beta-galactosidase binding protein (galectin-8) that binds with high affinity to a variety of cell surface integrins, thereby modulating cell adhesion and cell survival [42,43]. Adhesion between host and tumor cells, and extrinsic signals within the tumor microenvironment can promote an optimal niche for tumor cell survival and is an essential component of tumor invasion and metastasis [44,45]. New strategies for therapy have consequently been designed to disrupt these tumor-stromal cell interactions. For example, the inhibition of CXCR4 (a key receptor for tumor cell migration and adhesion) has been shown to overcome stromal-cell mediated drug resistance in acute myeloid leukemia and chronic lymphocytic leukaemia [46]. Clinical trials using specific integrin inhibitors have also shown promise in different types of solid tumours [47,48]. Clearly cell adhesion interactions have an important role to play in tumor progression; the observations from the present study indicate that they may also contribute to the mechanisms that lead to disease recurrence in ALL.

## Conclusions

We have used two different approaches to identify gene signatures that can successfully discriminate relapse and CCR patients at the time of diagnosis across multiple patient cohorts and platforms. Defined gene classifiers (such as the 5-GC) containing a smaller number of genes may be useful to augment existing risk stratification regimens for patients diagnosed with ALL as they can easily be adapted to qRT-PCR technology [18,22]. The complementary method we present here uses larger, biologically defined genesets that provide important clues to the underlying mechanisms of relapse. Such insights may provide for the development of improved therapies for T-ALL.

## Conflicts Of interests

The authors declare that they have no competing interests.

## Authors' contributions

ALC performed research, analyzed data, prepared manuscript; AHB directed research, analyzed data, prepared manuscript; MJF, RAO bioinformatics and statistical analysis; NCS performed research, collected data; SPH, provided specimens, revised manuscript; DLB designed study, provided specimens, revised manuscript; URK designed study, directed research, revised manuscript. All authors have read and approved the final manuscript.

## Supplementary Material

Additional file 1 Supplementary tables. Click here for file
