# Supplementary material for: Gene-based outcome prediction in multiple cohorts of pediatric T-cell acute lymphoblastic leukemia: a Children's Oncology Group study
Source: Mol Cancer. 2010 May 12;9:105. doi: 10.1186/1476-4598-9-105 (PMC2879253; doi:10.1186/1476-4598-9-105)
Supplement: Additional file 1 — Supplementary tables. [file 1476-4598-9-105-S1.DOC]

**SUPPLEMENTARY TABLES**

Table S1: 57 Probesets used for outcome prediction modeling in the training cohort.

| **RF order** | **Affy ID** | **Gene Symbol** | **R/CCR** |
| --- | --- | --- | --- |
| 6 | 217152_at | --- | 1.14 |
| 12 | 213497_at | ABTB2 | 1.31 |
| 17 | 219090_at | SLC24A3 | 1.19 |
| 20 | 221537_at | PLXNA1 | 1.16 |
| 21 | 218416_s_at | FLJ20489 | 1.15 |
| 29 | 206738_at | APOC4 | 1.12 |
| 31 | 222352_at | FBXO31 | 1.12 |
| 41 | 215541_s_at | DIAPH1 | 1.16 |
| 43 | 205798_at | IL7R | 0.55 |
| 47 | 211743_s_at | PRG2 | 1.60 |
| 51 | 208437_at | CLCN1 | 1.14 |
| 66 | 206881_s_at | LILRA3 | 1.17 |
| 84 | 203193_at | ESRRA | 1.12 |
| 100 | 208799_at | PSMB5 | 0.86 |
| 113 | 217881_s_at | CDC27 | 0.85 |
| 119 | 202797_at | SACM1L | 0.72 |
| 125 | 203878_s_at | MMP11 | 1.14 |
| 131 | 210018_x_at | MALT1 | 0.73 |
| 140 | 221652_s_at | C12orf11 | 0.80 |
| 142 | 202652_at | APBB1 | 1.14 |
| 147 | 222339_x_at | NFIX | 1.16 |
| 150 | 219041_s_at | REPIN1 | 0.65 |
| 157 | 209831_x_at | DNASE2 | 1.12 |
| 171 | 219118_at | FKBP11 | 0.74 |
| 183 | 202973_x_at | FAM13A1 | 0.76 |
| 195 | 218812_s_at | TMEM142B | 1.13 |
| 212 | 222265_at | TNS4 | 1.12 |
| 216 | 205848_at | GAS2 | 0.21 |
| 233 | 213601_at | SLIT1 | 0.68 |
| 245 | 220554_at | SLC22A7 | 1.14 |
| 247 | 205788_s_at | ZC3H11A | 0.81 |
| 249 | 204646_at | DPYD | 0.60 |
| 271 | 220451_s_at | BIRC7 | 1.13 |
| 288 | 208936_x_at | LGALS8 | 0.85 |
| 294 | 211433_x_at | KIAA1539 | 1.13 |
| 297 | 219014_at | PLAC8 | 0.57 |
| 301 | 208309_s_at | MALT1 | 0.72 |
| 306 | 206894_at | APOA4 | 1.12 |
| 318 | 203661_s_at | TMOD1 | 1.15 |
| 326 | 206945_at | LCT | 0.79 |
| 328 | 209730_at | SEMA3F | 1.15 |
| 329 | 221013_s_at | APOL2 | 1.12 |
| 356 | 217047_s_at | FAM13A1 | 0.83 |
| 357 | 201455_s_at | NPEPPS | 0.83 |
| 365 | 204658_at | TRA2A | 0.86 |
| 370 | 208654_s_at | CD164 | 0.76 |
| 381 | 216607_s_at | CYP51A1 | 0.66 |
| 385 | 215587_x_at | BTBD14B | 1.19 |
| 386 | 208405_s_at | CD164 | 0.81 |
| 387 | 216358_at | --- | 1.13 |
| 413 | 212159_x_at | AP2A2 | 0.70 |
| 429 | 206697_s_at | HP | 1.15 |
| 433 | 201801_s_at | SLC29A1 | 0.82 |
| 448 | 211779_x_at | AP2A2 | 0.72 |
| 456 | 217746_s_at | PDCD6IP | 0.86 |
| 477 | 209246_at | ABCF2 | 1.12 |
| 482 | 222252_x_at | UBQLN4 | 1.16 |

**Table S2: Cox proportional hazard regression analyses of the risk of relapse in the Validation Cohort (n=34) in relation to diagnostic features and 5-GC score; (A) multivariate; (B) univariate.**

|  | **Variable** | **No. of**  **Patients** | **Hazard Ratio** | **95% CI ^a^** | **p-value** |
| --- | --- | --- | --- | --- | --- |
| **A** | **Age at diagnosis** |  |  |  |  |
|  | < 10 years | 22 | 1 ^b^ |  |  |
|  | ≥ 10 years | 12 | 1.66 | (0.41, 6.81) | 0.48 |
|  | **WBC** |  |  |  |  |
|  | < 50/nl | 8 | 1 ^b^ |  |  |
|  | > 50/nl | 26 | 0.28 | (0.04, 1.88) | 0.19 |
|  | **Gender** |  |  |  |  |
|  | Female | 11 | * |  |  |
|  | Male | 23 | * | * | * |
|  | **5-GC score** | 34 | 3.76 | (1.40, 10.1) | 0.0088 |

| **B** | **Age at diagnosis** |  |  |  |  |
| --- | --- | --- | --- | --- | --- |
|  | < 10 years | 22 | 1 ^b^ |  |  |
|  | ≥ 10 years | 12 | 1.61 | (0.43, 6.01) | 0.48 |
|  | **WBC** |  |  |  |  |
|  | < 50/nl | 8 | 1 ^b^ |  |  |
|  | > 50/nl | 26 | 1.11 | (0.23, 5.33) | 0.9 |
|  | **Gender** |  |  |  |  |
|  | Female | 11 | * |  |  |
|  | Male | 23 | * | * | * |
|  | **5-GC score** | 34 | 2.94 | (1.16, 7.47) | 0.023 |

a) 95% confidence interval; b) reference group; * gender could not be assessed as no female relapsed.

**Table S3. GSEA leading edges from (A) NFκB pathway regulated genes, (B) Wnt/Ca2+/cGMP pathway, (C) cell adhesion receptor activity genesets.**

| **A** | **Probe Set ID** | **Gene Symbol** | **Gene** | **Cytoband** |
| --- | --- | --- | --- | --- |
|  | 205937_at | CGREF1 | Cell growth regulator with EF-hand domain 1 | 2p23.3 |
|  | 206128_at | ADRA2C | Adrenergic, alpha-2C-, receptor | 4p16 |
|  | 213038_at | RNF19B | Ring finger protein 19B | 1p35.1 |
|  | 216314_at | CRISP1 | Cysteine-rich secretory protein 1 | 6p21.3 |
|  | 221854_at | PKP1 | Plakophilin 1 (ectodermal dysplasia syndrome) | 1q32 |
|  | 222168_at | --- | --- | --- |
|  | 40420_at | STK10 | Serine/threonine kinase 10 | 5q35.1 |

| **B** | 201187_s_at | ITPR3 | Inositol 1,4,5-triphosphate receptor, type 3 | 6p21 |
| --- | --- | --- | --- | --- |
|  | 202660_at | ITPR2 | Inositol 1,4,5-triphosphate receptor, type 2 | 12p11 |
|  | 203710_at | ITPR1 | Inositol 1,4,5-triphosphate receptor, type 1 | 3p26-p25 |
|  | 206623_at | PDE6A | Phosphodiesterase 6A, cGMP-specific, rod, alpha | 5q31.2-q34 |
|  | 210304_at | PDE6B | Phosphodiesterase 6B, cGMP-specific, rod, beta | 4p16.3 |
|  | 210404_x_at | CAMK2B | Calcium/calmodulin-dependent protein kinase (CaM kinase) II beta | 22q12 / 7p14.3-p14.1 |
|  | 211093_at | PDE6C | Phosphodiesterase 6C, cGMP-specific, cone, alpha | 10q24 |
|  | 212128_s_at | DAG1 | Dystroglycan 1 (dystrophin-associated glycoprotein) | 3p21 |
|  | 212757_s_at | CAMK2G | Calcium/calmodulin-dependent protein kinase (CaM kinase) II gamma | 10q22 |
|  | 214064_at | TF | Transferrin | 3q22.1 |
|  | 215092_s_at | NFAT5 | Nuclear factor of activated T-cells 5, tonicity-responsive | 16q22.1 |
|  | 216883_x_at | PDE6D | Phosphodiesterase 6D, cGMP-specific, rod, delta | 2q35-q36 |

| **C** | 201124_at | ITGB5 | Integrin, beta 5 | 3q21.2 |
| --- | --- | --- | --- | --- |
|  | 201474_s_at | ITGA3 | Integrin, alpha 3 | 17q21.33 |
|  | 204625_s_at | ITGB3 | Integrin, beta 3 | 17q21.32 |
|  | 204990_s_at | ITGB4 | Integrin, beta 4 | 17q25 |
|  | 205032_at | ITGA2 | Integrin, alpha 2 (CD49B, α2 subunit VLA2 receptor) | 5q23-q31 |
|  | 205422_s_at | ITGBL1 | Integrin, beta-like 1 (with EGF-like repeat domains) | 13q33 |
|  | 205785_at | ITGAM | Integrin, alpha M | 16p11.2 |
|  | 206009_at | ITGA9 | Integrin, alpha 9 | 3p21.3 |
|  | 206025_s_at | TNFAIP6 | Tumor necrosis factor, alpha-induced protein 6 | 2q23.3 |
|  | 206766_at | ITGA10 | Integrin, alpha 10 | 1q21 |
|  | 212128_s_at | DAG1 | Dystroglycan 1 (dystrophin-associated glycoprotein 1) | 3p21 |
|  | 205816_at | ITGB8 | Integrin, beta 8 | 7p15.3 |
|  | 215879_at | ITGB1 | Integrin, beta 1 | 10p11.2 |
|  | 216331_at | ITGA7 | Integrin, alpha 7 | 12q13 |
